# Who cares for the carer? Codesigning a carer health and wellbeing clinic for older care partners of older people in Australia

**DOI:** 10.1111/hex.13863

**Published:** 2023-09-08

**Authors:** Natasha Layton, Aislinn Lalor, Susan Slatyer, Den‐Ching A Lee, Christina Bryant, Moira Watson, Anjali Khushu, Elissa Burton, Déborah Oliveira, Natasha L. Brusco, Alessandro Jacinto, Elizabeth Tiller, Keith D. Hill

**Affiliations:** ^1^ Rehabilitation, Ageing and Independent Living (RAIL) Research Centre Monash University Frankston Victoria Australia; ^2^ National Centre for Healthy Ageing Monash University and Peninsula Health Frankston Victoria Australia; ^3^ Department of Occupational Therapy, School of Primary and Allied Health Care Monash University Melbourne Victoria Australia; ^4^ Centre for Healthy Ageing Murdoch University Murdoch Western Australia Australia; ^5^ Melbourne School of Psychological Sciences University of Melbourne Melbourne Victoria Australia; ^6^ Department of Geriatric Medicine Peninsula Health Frankston Victoria Australia; ^7^ Curtin School of Allied Health Curtin University Bentley Western Australia Australia; ^8^ enAble Institute, Faculty of Health Sciences Curtin University Bentley Western Australia Australia; ^9^ Faculty of Nursing, Universidad Andrés Bello Campus Viña del Mar Viña del Mar Chile; ^10^ Millennium Institute for Care Research (MICARE) Santiago Chile; ^11^ Programa de Pós‐Graduação em Medicina Translacional, Faculty of Geriatric Medicine Federal University of São Paulo (UNIFESP) São Paulo Brazil; ^12^ Department of Geriatrics (Falls Prevention Service) Peninsula Health Frankston Victoria Australia

**Keywords:** carer, clinic, health, model of care, partnership, wellbeing

## Abstract

**Introduction:**

Older carers or ‘care partners’ of older people experiencing care needs often provide essential support, at times while neglecting their own health and well‐being. This is an increasingly frequent scenario due to both demographic changes and policy shifts towards ageing in place. Multiple community stakeholders within the care and support ecosystem hold valuable expertise about the needs of older care partners, and the programme and policy responses that may better support their health and well‐being. The aim of this study was to identify the perspectives of stakeholders obtained through the codesign phase of a multicomponent research project investigating new models of care and support for older care partners suitable for the Australian context.

**Methods:**

Principles of codesign were used to engage a purposeful sample of older care partners, health professionals, researchers, policy makers and health service administrators. Participants took part in a series of three codesign workshops conducted remotely via video conferencing. The workshops were supported with briefing material and generated consensus‐based summaries, arriving at a preferred service model.

**Findings:**

This paper reports the research design and structure of the codesign panels, the range of findings identified as important to support the health and well‐being of older carers of older people, and the resulting service model principles. The codesigned and preferred model of care is currently being prepared for implementation and evaluation in Australia.

**Public Contribution:**

This study was conducted using codesign methodology, whereby stakeholders including older care partners and others involved in supporting older carers, were integrally involved with design, development, results and conclusions.

## 1 INTRODUCTION

Many older people live with chronic conditions and rely on the care of both family members and/or friends (who often are older people themselves) and community services to support them to continue to live at home. The term carer or care partner is used to describe individuals who take on a supportive care role with day‐to‐day activities and health care needs.^1^ Both terms are used interchangeably throughout this paper. While some positive aspects of providing care have been reported by care partners, including a sense of giving back or reciprocity (e.g., in the case of caring for a parent), and as a continued part of a loving relationship (where this was the relationship status before commencing the carer role)^1^, ^2^, ^3^ providing care can have detrimental effects on the health and well‐being of care partners including emotional stress, physical strain, exhaustion, and loneliness.^4^, ^5^, ^6^ These negative consequences of the caregiving role can lead to reduced focus on the care partner's own health, and increased risk of negative health outcomes, such as depression and anxiety.^7^ Developing and implementing effective multidisciplinary approaches to promote care partners' health is likely to have positive effects on their health and well‐being, as well as ensuring the sustainability of the care partner role.^5^, ^8^ They can also have positive impacts on health and care systems through delayed institutionalisation or reduced hospital use by care recipients.^9^ In the United Kingdom, an approach has been instituted whereby care partners have a statutory right to have their needs assessed in addition to the assessments routinely provided for the people for whom they care.^7^ However, there is little evidence in Australia that services currently provide a comprehensive needs‐based assessment and tailored support to ensure that care partners' health and well‐being are protected from the negative effects of their caring role. Developing and implementing a specialised care partner health service is, therefore, a national public health priority.

An important further consideration is the dynamic nature of caregiving. Moving into and moving through a care partner relationship is termed ‘situational transition’.^10^ Often termed ‘caregiving’, these care partner transitions have been characterised as ‘successful’, ‘challenging’, and many gradations in between. Multiple factors influence outcomes for the care partner and for the dyad (care partner and care recipient). Factors that facilitate or inhibit this transition include previous caregiving experience, personal spirituality and beliefs, the presence or absence of family support networks, health services and financial stressors. Multiple commitments, emotional and physical health conditions, and advanced age are also factors. For example, spousal care relationships can be influenced by the quality of the spousal relationship before care partner–care‐recipient roles coming about. Johansson et al.^11^ suggest that for the positive value of care partner relationships to be enhanced and the negative impact reduced, different aspects of the relationship need to be recognised and addressed.

Much carer research includes care partners of all ages. Informal carers (care partners) are defined as ‘a family member/s and/or friend/s who routinely support the older person through assistance with household tasks, self‐care and mobility, emotional and social support, treatments, medication, responding to acute health needs, advocacy and care coordination, or surrogate decision‐making’.^12^ In Australia, almost half (47%) of all care partners are over the age of 55 years.^13^ Older care partners, for the purposes of this study, are defined as being aged 50 years or older. Older care recipients are defined as people over the age of 65, reflecting commonly used cut‐offs where increasing health problems and comorbidities contribute to a growing need for assistance for independent living.^9^ While there are some generic factors affecting health and well‐being of care partners of all ages, there are additional factors and potentially different intervention approaches that will be more effective and relevant for older carer partners. Additionally, even mild physical or mental health conditions developing in care partners in this age group may be complicated by the requirements and mental and physical health demands associated with caring roles.^5^

Caring for an older person experiencing care needs is usually a progressive role in which care responsibilities increase in number and complexity over time.^14^ For care partners who are older people themselves, the challenges related to their health are an additional concern.^14^ These challenges are further exacerbated by current trends, such as shorter hospital stays and out of hospital health care, which is fragmented and difficult to navigate for older people and their care partners. These factors compound to shift the burden of support from acute care settings to patients and their social networks (often care partners) in the community.^15^

Much of the existing research investigating care partner needs and interventions focusses on care partners of people with specific health conditions, such as care partners of people with dementia.^16^, ^17^ Focusing on care partners of people with a particular health condition provides a basis to target specific unique needs associated with caring for a person with these conditions that may not be as important or relevant to care partners of people with other health conditions. For example, resources and stressors were found to be different in care partners caring for people with dementia, Parkinson's and cancer.^18^

From an intervention perspective to improve health and well‐being outcomes for care partners, there is growing research evidence of effective approaches. These include single‐intervention approaches evaluated in randomised controlled trials reporting positive health and well‐being outcomes for care partners from psychosocial and education programmes,^19^, ^20^ physical activity or exercise programmes,^21^, ^22^, ^23^, ^24^ occupational therapy programmes,^25^ goal setting,^26^ respite care^27^ and psychoeducation intervention.^28^ Multicomponent interventions that combine two or more of these approaches have also been shown to be effective.^29^, ^30^, ^31^ Despite this research, there is very little evidence of translation of these approaches into routine practice. Furthermore, few of these studies have focussed on older care partners of older people—a particular subgroup of care partners with specific needs and challenges, that is the focus of this study.

It is likely that older care partners, and in particular those providing care for older people, have unmet service needs. Our review of the literature did not identify many health service responses geared to older care partners. The only identified model for a multidisciplinary health service providing assessment and interventions to address unmet needs for informal care partners aged >55 years was located in Sao Paulo (Brazil).^32^ This master's thesis reported the profile of carers attending the service (the team included a social worker, psychologist and physician), who provided assessment and interventions focusing on the medical, social and psychosocial aspects of carers' needs. No formal evaluation of the outcomes of this novel service have been conducted. Given the novelty of this service approach in Sao Paulo, members of our research team have previously visited this service, and collaborated with two Brazilian researchers (A. J. and D. O.) (one associated with this service) on the current project, including undertaking interviews with staff at that service.

The aim of this study was to integrate the voice of the care partner and other stakeholders in service design in determining a preferred service model to improve the health and well‐being needs of older care partners of older people. Individual stakeholders may have different perspectives about knowledge. Seeking multiple perspectives about a particular topic supports ‘strong objectivity’.^33^ This is particularly the case in hearing from individuals who know about and experience services yet do not hold positions of power and dominance. This type of experiential knowledge about health services is recognised as pivotal to policy and service design^34^, ^35^ as well as to research.^36^ Codesign is a strategy to collaboratively design services with service‐users, service‐deliverers and service‐procurers. A substantive evidence base underpins the philosophy of consumer involvement and the practice of codesign across fields including public health,^37^, ^38^ social care^39^ and community development.^40^ Some codesign approaches are intended for use across multiple fields and contexts and there is no gold‐standard approach.^41^, ^42^ Results from this study will inform planning, development and evaluation of a new intervention model for improving the health and wellbeing of older care partners of older people through the use of stakeholder codesign.

## 2 MATERIALS AND METHODS

This qualitative study utilised codesign principles and frameworks within focus‐group methodology. Toolkits designed for the Australian community and disability sector were used to guide the codesign process used in this study.^43^, ^44^

### 2.1 Participants

Stakeholders included those with lived experience of being an older care partner, and professionals involved in providing health care, administering health services or policy, or researching with carers or older people. The purposeful selection strategy involved multiple targeted groups and avenues, including: (1) carer associations, state departments of health, research facilities addressing ageing and specialist geriatric clinics; and (2) individuals who had been working in, publishing in, or otherwise contributing to the area of caring for carers.

Consistent with the codesign methods selected, steps were taken to build equity and inclusion into the selection of a cross‐section of voices. Two of the research team reviewed and prioritised invitations according to three principles: (1) diversity (conceptualised as spanning disability, gender, culture and linguistic diversity); (2) equity (i.e., mapping and prioritising underserved communities likely to utilise care partner clinics within their catchment); and (3) intersectionality lens (by recognising that older care partners themselves may be informed by previous roles related to aged care, and that many professionals also had experiences of care partner journeys within their own family networks). Sixteen participants were included in the codesign panel, broadly balanced across four categories (Table 1).

**Table 1:** Stakeholder mix of codesign panel.

Makeup of the codesign panel	Diversity and equity filter
Older care partners (>50 years) of older people (>65 years)	Multiple genders From three Australian states Rural/urban Current care partners Past care partners (who were bereaved or had relinquished care) Multiple cultural backgrounds Experience of disability Experience of age‐related issues
Health professionals	
Policy and planning staff	
Health service administrators	
Academic researchers	

### 2.2 Setting

Due to Covid‐19 restrictions, on‐line video conferencing (Zoom) was utilised throughout, with participants across three time zones within Australia.

### 2.3 Recruitment and consent

Once stakeholders (care partners, health professionals, policy and planning staff, health service administrators and researchers) were identified, one of the research team sent an email invitation with the explanatory statement and consent form. All consent forms were collected and stored electronically before commencement of the first codesign panel. No reimbursement was offered other than a recognition of $AUD200 in gift vouchers per panel meeting (including preparation and panel meeting time) for participants whose work roles did not cover their time on the panel, which included care partner participants.

### 2.4 Data sources

This codesign project was the final phase of a larger mixed method study (‘Who cares for the carer?’) aiming to understand the needs of older care partners of older people in relation to health, well‐being, health promotion, access to services and service requirements for the future. Other study components included (i) a survey of older care partners of older people^45^; (ii) interviews of older care partners of older people and health professionals who worked with older care partners of older people across multiple sites in Australia (manuscript under preparation); and (iii) targeted interviews with the Sao Paulo Carer Clinic staff in Brazil.

1. Carer surveys (*n* = 189 older care partners)^45^ and carer interviews (*n* = 14 older care partners) (under preparation) included demographic information about the care partner, their caring role, self‐reported health and well‐being, and the avenues they pursued to optimise their own health and well‐being, including when seeking health advice for the person for whom they cared. Additionally, they were asked about any factors that influenced the uptake of practices to improve their own health, as well as recommendations they may make to help improve their own health and well‐being and sustain their caring role in the longer term.
2. Health professionals' interviews in Australia (*n* = 21) and Brazil (*n* = 4) collected information about the nature of advice and support provided to older care partners about their own physical and mental health, strengths and weaknesses of existing services in supporting the health and well‐being of older carers of older people, and suggestions for improved or alternative approaches to improve longer‐term care partner health and well‐being.

**Figure 1:**
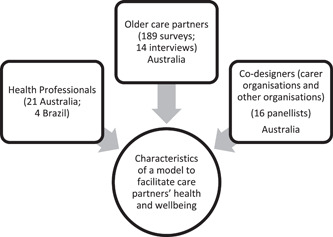
Knowledge sources informing a carer support model.

Key findings from these datasets and from the literature were synthesised into short briefing notes containing plain language summaries about (a) the current evidence base, (b) challenges and (c) potential solutions. Each set of briefing notes was emailed to codesign panellists 1 week before each panel. The intent was to provide all panellists with a broad scope of information and ideas to draw upon while exploring and conceptualising a model of care for older care partners of older people. The agenda of each panel contained questions and discussion points, which were led and live annotated by one of the research team into PowerPoint, enabling panellists to guide, edit and achieve consensus around these points. The virtual focus groups were recorded with permission and transcribed verbatim (www.wordism.com.au). The transcriptions and PowerPoint annotations were read and summarised by a research team member who created panel summaries that were member‐checked by at least two authors, and then circulated to all panellists for verification. Appendix A details the briefing packs provided to panellists. Panellists were invited to correspond with the research team via email or phone call between sessions for clarification as required. Figure 1 illustrates the range and diversity of stakeholder input, totalling 243 voices across older care partners, health professionals and codesigners.

### 2.5 Procedure

**Figure 2:**
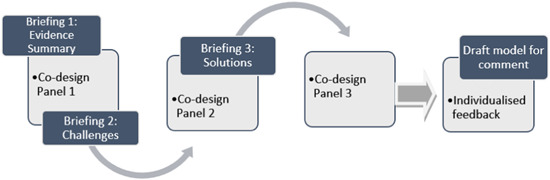
Codesign panel process.

The codesign panel met three times via a Zoom, approximately 3 weeks apart over 8 weeks from May to June 2022. Each panel lasted 90 min (see Figure 2).

A range of strategies were utilised to facilitate the codesign principle of combining wisdom drawn from lived experience with the expertise of professionals, to manage any power disparities and to maximise benefit.^44^, ^45^ The briefing notes and introductory remarks in each panel included an explicit commitment to the equal status of all panellists. Panel facilitators worked to enact and enforce principles of collaboration and respect through supportive questioning across panellists, and a check‐in via phone or email was made to the panel members who were older care partners, between panel meeting 1 and panel meeting 2. Meeting 2 incorporated breakout groups in Zoom where older care partners had the opportunity for small group discussion with two researcher facilitators, and other panellists were in separate groups. Sessions were tailored to be culturally safe (inclusive of acknowledgement of country, convenient time periods established via online polling). The research team invited individual dialogue, with communication channels being email and phone for written or verbal feedback during or between sessions. Appendix B contains the panel agenda.

### 2.6 Data analysis

Both thematic coding and an extant coding framework were applied to panel transcriptions. The coding framework was a subset of the World Health Organisation's International Classification of Functioning, Disability and Health (ICF) domains, including activities and participation and environmental factors (natural and built environment, products and technology, support, relationships and attitudes, services, systems and policies).^46^ This coding framework was selected based on the evidence base recommending broad socioecological views of barriers and facilitators.^47^ This international classification, adopted within Australia's health systems, is highly relevant to specifying both the areas of care partner need and the comprehensive structures that may need to be included in developing a new model of care for older care partners.^48^

Once coded, results were organised into a series of panel summary documents. The research team independently reviewed these, meeting via Zoom to arrive at consensus on each panel summary. Panel participants had the opportunity to edit or respond to the summaries as a member‐checking step, however no edits were received.

The panel summaries became units of analysis for the final phase, where the research team analysed the combined summaries along with briefing notes to articulate the emerging principles for a model of care for older care partners. This draft model was then shared via email with panellists for comment.

## 3 RESULTS

Sixteen stakeholders took part (Table 2), along with three members of the research team. Thirteen panellists attended all three panels and three panellists attended two panels. Where participants did not attend, this was due to logistical issues or unplanned care partner commitments, and in each instance, contact was made with the research team to provide an apology or provide written or verbal input based on the briefing notes. Communication (phone calls and emails) was received from a total of six panellists, to share resources and provide additional points to be recorded.

**Table 2:** Codesigners and panel attendance.

Codesigners	Panel attendance (total numbers at each panel)		
	Panel 1	Panel 2	Panel 3
Older care partners (3) Victoria, Queensland, WA.	3	3	2
Health service administrators (3).	3	3	3
Health professionals providing health care services to older people with older care partners (1).	1	1	1
Consumer/stakeholder organisations (4) (carer organisations; dementia organisations).	4	4	4
External researchers (4) (three universities from three states; three disciplines).	2	4	3
Total	13	14	13

Four themes were derived from the data generated through the codesign panels: (1) naming and framing; (2) outcomes which a carer support model might deliver; (3) the ecosystem; and (4) what might be delivered and who might deliver it. The themes are described in more detail below.

### 3.1 Theme 1: Naming and framing

A range of perspectives were shared around language and the positioning of the model in relation to health or broader constructs. ‘Care partner’ was strongly suggested by participants in care partnerships, as a term that embeds more empowerment or equality. The terms ‘clinic’ and ‘model of care’ were discussed. Respondents who were care partners identified the way existing medical paradigms, expressed through terms such as ‘referral’, ‘prescription’, ‘clinic’, shaped expectations of a service. The term ‘clinic’ was thought to be widely understood and a useful heuristic for a model that brings together valued supports for care partners. Some participants were concerned the term ‘clinic’ concept was overly clinical and neglected social domains. Active signalling was recommended to indicate that a clinic will address needs for care partners ‘beyond health’ and include, for example, collaboration, education and planning support.

### 3.2 Theme 2: The outcomes a carer support model might deliver

Panellists identified inputs that would be required at multiple levels to achieve outcomes. Health evaluation and interventions were considered to be the starting point for older care partners. Counselling and psychological support targeted at sustaining the care partnership but working from the perspective of the care partner were seen as a further priority. This could include links to respite services and tertiary referral, along with deep listening to the situations of individual care partners and linking them into appropriate interventions such as counselling and financial services. Allied health professional inputs were proposed to address environments, products and technology. For example, dietetics to support nutrition, occupational therapy and physiotherapy to provide relevant products and address environmental barriers and exercise options, and social work to support the care dyad to access services, systems and policies.

### 3.3 Theme 3: The ecosystem

**Figure 3:**
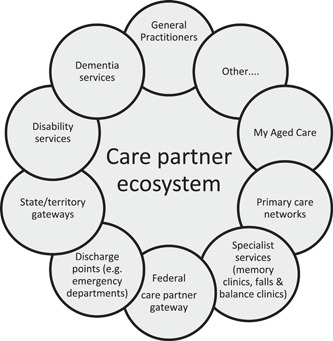
Care partner ecosystem.

Codesigners envisioned a model that does not duplicate other services within the current network or ecosystem of services and resources. Additionally, any model must be culturally safe and culturally appropriate and ensure that any offerings to care partners are feasible. For example, considering the need for respite to be able to be organised for a care recipient so that the care partner is able to engage fully with the service. The model will be informed by good existing practices and avoid pitfalls noted from similar initiatives (see Figure 3).

### 3.4 Theme 4: What might be delivered and who might deliver it?

The pragmatics of meeting need were comprehensively discussed and core ingredients of a model identified. An ideal service was considered a Carer Health and Well‐Being Clinic designed to be a centre of practice excellence, which would provide responsive and timely support, information, services and interventions for older care partners of older people, based on care partner needs and priorities, and evaluation of these. It was seen as important to focus on ‘well‐being’ as well as ‘health’. While the term ‘clinic’ was felt to be a medical one, it was felt to be less ambiguous than ‘service’. An active networking approach to link to existing supports was seen as key to the establishment of such a clinic. This would entail proactively building links with the local networks, including local government resources, local health networks, state and commonwealth resources, and state‐based carer associations. Associated with this is the strategic development of digital access (establish a user‐friendly website, deliver ongoing updates to website, e.g., links to care partner organisations' websites, relevant health pathways, relevant resources and information).

The physical location of such a service may be within a health service (easy linkage with some other relevant services) or be located within a community setting (perhaps a community health centre or other centre providing health and wellbeing services within a local community). Operationally it was proposed to operate 2 days/week (minimum—though this would be funding dependent), offering hybrid service delivery (i.e., centre‐based, in home and virtual). A core activity would entail communication (with care partner permission) with existing service providers, such as general practitioners.

The staffing model was recommended to be multidisciplinary. Disciplines considered to potentially have valuable roles included a clinic manager (e.g., nurse/counsellor), social worker/counsellor, psychologist or clinical psychologist, physiotherapist, occupational therapist, dietitian and a peer support role/peer support coordinator). Two staffing models were put forward: (1) a key worker model, whereby each staff member has a case management load matched to their skillset, with additional staff engaged as needed; or (2) individual staff assessments based on intake assessment and carer‐determined priorities, with team meetings or documentation to share findings and recommendations.

Assessment was envisaged to include, first, a minimum data set which maximises useful data for evaluation of outcomes, while minimising demands on care partners' time. Second, comprehensive, individualised assessment. This may entail a suite of standardised tools suitable for older care partners relevant to the selected intervention/s targeted for each carer (e.g., measures may include assessment tools for depression, anxiety or care partner burden for those with psychological concerns). These would provide a baseline measure and identify any changes for the purposes of evaluating the pilot clinic, as well as identifying potential areas for action and change with the care partner by utilising outcomes of the psychosocial, physical, nutritional, activity and quality‐of‐life screening.

The service model was envisaged as providing individualised support at critical points in the care journey. It was considered important to recognise and meet the needs of carer diversity (including culturally and linguistically diverse communities). A strengths‐based capability‐building approach was recommended, to enable carers to become peer supporters if desired. Some strategies to deliver individualised support included:

1. Flexible entry points (self‐referral, able to respond to warm leads from other services, where carer needs were identified, for example, general practitioners, ongoing ability to re‐enter service as needs change over time);
2. Practical session options (e.g., in‐home respite needs whilst care partner attends, offer telehealth options if preferred; proactive work to set up suitable arrangements);
3. Streamlined service offerings for individuals or for groups. Ideas included topics such as (i) Future proofing my own health (fitness screen, manual handling, environmental review, exercise options, including home exercise programmes with intermittent therapist oversight and review); (ii) *Rolling with the punches* (balancing my own needs with those of the person I provide care for, setting goals, group peer support sessions, intermittent phone reviews); (iii) *Building my resource kit* (social, financial and family‐related discussions with counsellor and relevant resources—may include referral/recommendation to seek legal, financial, family dynamics advice and guidance—and connect with peer supporter, optional group sessions, intermittent phone reviews); and (iv) *Building my knowledge* (multimodal, including group, individual, online, hard copy information), include peer education sessions.

The sustainability of a Carer Health and Well‐Being Clinic was also highlighted, noting economic and programme evaluation will be an essential step in establishing and evaluating such a clinic.

## 4 DISCUSSION

This paper reports the outcomes of a codesign panel process to make recommendations on key elements of a preferred new service model (a Carers Health and Well‐Being Clinic) to improve health and well‐being outcomes for older care partners of older people. The panels brought together key stakeholders, including older care partners of older people, representatives from carers' organisations and other relevant consumer organisations (e.g., state‐based care‐partner and dementia organisations), health service administrators, health professionals, government policy personnel and researchers. The panel meetings were facilitated to ensure all participant voices were heard and an iterative process occurred across the three panel meetings to develop a preferred model for an innovative new service to address gaps identified by our previous research^45^ and that of others.^8^, ^49^

There was general consensus across the panel participants for the preferred structure and function of the proposed Carer Clinic. The preferred elements of flexible entry point, streamlined services, practical sessions and education, as described in the results, form a framework of principles for a new service to improve health and well‐being outcomes for older care partners, although a final model in terms of scale, staffing, associated services and interventions able to be delivered directly through the clinic versus those requiring referral to other services, are dependent upon funding obtained to implement and evaluate the Carer Clinic model.

Our team has only identified one dedicated Carer Clinic service globally through literature searching and drawing on the knowledge of grey literature (a master's thesis).^32^ This out‐patient service was established in 2007 in Sao Paulo, Brazil. The service targeted carers aged >55 years of age and provided multidisciplinary assessment and interventions through a team consisting of a social worker, psychologist and physician. The focus of the service was on medical, social and psychosocial aspects of care partner needs, which differs from the broader health focus from previous research literature described above and our own findings from interviews with Australian care partners.^45^ The master's thesis reported interviews with 48 care partners (mean age 66 years) attending the service, mainly reporting satisfaction with the service and benefits perceived to be associated with clinic attendance over an average period of 3 years interaction.^32^

The envisioning of an ideal model as has resulted from this project may be of use in formulating real service options and research projects to address the identified unmet needs of older care partners of older people. The codesign process provides a blueprint and narrative against which to design and evaluate any new service offering. In summary, such a clinic would focus on health promotion and address individual care partner needs to maintain the care partner's health and well‐being long term, maximising the likely sustainability of the care partner role and, thereby, the older care recipient remaining at home. From a service delivery perspective, there can be common elements in the caring role (e.g., the need for information or respite), as well as the unique needs of care partners arising from the health condition of the care recipient, and also the personal needs and preferences of the individual care partner that need to be considered.^5^, ^50^ Therefore, a flexible model of a ‘one‐stop‐shop’ to meet care partner support and care partner health needs and provide avenues of support for care partners of people with specific health conditions may best meet care partners' generic and specific needs. This would address the dual goals of supporting care partners' long‐term health and well‐being, as well as sustaining the caring role. These principles are echoed in recent work directly with older care partners.^49^ A recently published integrative review of health promoting behaviours developed by informal caregivers of older adults reinforces the codesign panel findings regarding the broad range of resources needed for the maintenance of their overall health, well‐being, the improvement of quality of life and personal satisfaction.^49^ Among the needs carers identified that they wished to access, the search for information, family counselling, social, emotional and spiritual support stood out. Also, engagement with community programmes aimed at psychoeducation and physical activities, as well as the use of communication technologies and the enhancement of self‐care were described as strategies associated with coping with the potential negative repercussions resulting from caring.

## 5 STRENGTHS AND LIMITATIONS

The study had a number of strengths associated with use of codesign panel methods, including adherence to a formalised Australian codesign process.^43^ Panels explicitly established codesign groundwork with inclusive, first‐person language, and the framing of practice‐based knowledge or expertise‐by‐experience as valued and equal to other forms of knowledge. A range of engagement strategies, including written and presented briefing packs, live mapping and breakout groups for in‐depth discussions, with mailed summaries and the integration of feedback, aimed to provide transparent processes by which the panels formed consensus positions on a preferred model of service for older care partners of older people. The high degree of panel attendance and incidental communication between panels demonstrated a high degree of commitment to the process. In contrast, some alternative codesign approaches separate stakeholder groups as a way to manage power differences and to build confidence in speaking to ‘like‐others’.^51^ We utilised breakout groups for this purpose in Panel 2 and this experience, according to two of the older care partners, did increase confidence in contributing. However, a limitation is that views obtained will not be exhaustive. Some participants were embedded in their state or jurisdictional contexts which gives a particular lens on what is feasible. The research context (scope was confined to older care partners of older people) itself sets some boundaries or limitations on a comprehensive exploration of options, although we consider there to be a strong rationale provided for targeting older care partners of older people.

## 6 CONCLUSIONS

This paper reports on a codesign process and the preferred model of service to meet the needs of older care partners of older people in Australia. Codesign credentials were built into our method, and the multiple data sources from a related local survey, interviews, and the current evidence base provided a strong foundation for the multiple codesign panel deliberations. We suggest the resulting principles for a service to support the health and well‐being of older care partners of older people will be of value in conceptualising and implementing service reform to meet current and future needs.

## AUTHOR CONTRIBUTIONS

All authors have made a significant contribution to the work reported, whether it is the conception, study design, execution, acquisition of data, analysis and interpretation, or in all these areas. Natasha Layton wrote the article. All other authors have substantially revised or critically reviewed the article. All authors have agreed on the journal to which the article is submitted, reviewed, agreed on all versions of the article before submission, during revision, the final version accepted for publication, and any significant changes introduced at the proofing stage, and agreed to take responsibility and be accountable for the contents of the article.

## CONFLICT OF INTEREST STATEMENT

The authors declare no conflict of interest.

## ETHICS STATEMENT

The study was conducted according to the guidelines of the Declaration of Helsinki, and approved by the Research Ethics Committee of Monash University (27379) on 30/03/2021. Informed consent was obtained from all subjects involved in the study.

## Data Availability

The data that support the findings of this study are available on request from the corresponding author. The data are not publicly available due to privacy or ethical restrictions.

## APPENDIX A SUMMARY OF MATERIALS PROVIDED TO CODESIGN PANELS

**Table:**

*Briefing pack 1 (provided 1 week before Panel 1)*	
Information about the purpose, process and scope of the codesign consultations	Explanatory statement, and email containing agenda explicitly states: *you are invited to be part of a codesign panel involving older care partners of older people, health professionals providing health care services to older people with older care partners, health service administrators, policy and planning staff and researchers.*
Evidence summary	Brief plain language summary of the research evidence of what has been shown to be effective in improving health of older care partners (e.g., physical activity programmes, psychoeducational approaches). This includes emerging research areas (that may not have randomised controlled trial or systematic review level evidence, but have indications of promising outcomes that warrant further research).
*Briefing pack 2 (provided 1 week before Panel 2)*	
Summary of Panel 1	Source: Transcribed and summarised Panel 1 proceedings and PowerPoint slide pack, including live‐captured discussion points.
Briefing note: Voices of care partners and health professionals about issues and challenges	Sources: Care partner surveys; care partner interviews (details reported in separate paper.^45^
*Briefing note 3 (provided 1 week before Panel 3)*	
Summary of Panel 2	Source: Transcribed and summarised Panel 2 proceedings and PowerPoint slides, including live‐captured discussion points.
Briefing Note: Solutions from carers; solutions from health professionals	Sources: Care partner survey; care partner interviews (details reported in separate paper,^45^ under review).
*Briefing pack 4 (provided 3 weeks after Panel 3)*	
Panel 3 summary	Source: Transcribed and summarised Panel 3 proceedings and PowerPoint slide pack, including live‐captured discussion points.
Carer clinic model: Draft for comment from codesigners	Researcher‐led synthesis of codesign panel deliberations regarding a preferred model for caring for older carers of older people (requesting feedback from panel).

## APPENDIX B CODESIGN PANEL AGENDAS

**Table:**

Panels	Agenda
Panel 1: ‘what is needed’?	1. Welcome 2. Introductions 3. Overview of project and codesign panel activities 4. What the published evidence tells us 5. Our lived experience evidence and our practice knowledge ‘What needs are unmet’ ‘Wish list’ activity 6. Checking in: − any questions and comments about the scope of the codesign − looking ahead to where we need to end up at panel
Panel 2: Needs and barriers	1. Welcome and introduction to new panellists 2. Any comments on summary of Panel 1? 3. Voices of care partners and health professionals (prereading pack) 4. Live capture of panel reflections and additions 5. care partner journey mapping ‘what service journeys COULD look like’ (*small groups*) 6. Checking in. Preparation for final panel
Panel 3: ‘What would good look like’	1. Welcome and reflections on panels to date 2. Panel 3 focus on solutions *supported by briefing notes: care partner codesign Panel 3 summary care partner codesign briefing note Panel 3 solutions from health professionals care partner codesign briefing note Panel 3 solutions from care partners 3. What does ‘good’ look like: Guided discussion and feedback 4. Next steps 5. Close and thanks
